# Differential effects of bariatric surgery on circulating GDF15 and FGF21 levels: Implications for glycemic status and weight loss outcomes

**DOI:** 10.1111/eci.70069

**Published:** 2025-05-16

**Authors:** Laura Salmón‐Gómez, Victoria Catalán, Beatriz Ramírez, Maite Aguas‐Ayesa, Amaia Rodríguez, Sara Becerril, Víctor Valentí, Rafael Moncada, Carolina M. Perdomo, Camilo Silva, Javier Escalada, Gema Frühbeck, Javier Gómez‐Ambrosi

**Affiliations:** ^1^ Metabolic Research Laboratory Clínica Universidad de Navarra Pamplona Spain; ^2^ Centro de Investigación Biomédica en Red‐Fisiopatología de la Obesidad y Nutrición (CIBEROBN) Instituto de Salud Carlos III Pamplona Spain; ^3^ Obesity and Adipobiology Group Instituto de Investigación Sanitaria de Navarra (IdiSNA) Pamplona Spain; ^4^ Department of Endocrinology & Nutrition Clínica Universidad de Navarra Pamplona Spain; ^5^ Department of Surgery Clínica Universidad de Navarra Pamplona Spain; ^6^ Department of Anesthesia Clínica Universidad de Navarra Pamplona Spain

**Keywords:** excess weight loss, FGF21, GDF15, obesity, roux‐en‐Y‐gastric bypass, type 2 diabetes

## Abstract

**Background:**

Type 2 diabetes (T2D) is a comorbidity commonly associated with obesity. Elevated concentrations of growth differentiation factor 15 (GDF15) and fibroblast growth factor 21 (FGF21) are associated with these conditions, making both cytokines interesting candidates to combat them. This study aimed to analyse the relationship between changes in plasma GDF15 and FGF21 levels and the resolution of T2D or obesity improvements after bariatric surgery.

**Methods:**

Plasma samples from 104 patients (52 with obesity and normoglycemia and 52 with obesity and impaired glucose tolerance or T2D) were analysed before and after Roux‐en‐Y‐gastric bypass surgery.

**Results:**

Plasma GDF15 levels increased significantly after bariatric surgery in patients with obesity and normoglycemia (*p* < 0.01), as well as in those with obesity and impaired glucose tolerance or T2D (*p* < 0.05). This increase was significant in patients analysed up to 8 months after surgery in both groups (*p* < 0.01) but not in those analysed between 8 to 15 months after surgery, suggesting that GDF15 concentrations exhibit an early increase after surgery but may return to baseline levels over time. In contrast, plasma FGF21 levels after bariatric surgery decreased significantly in patients with impaired glucose tolerance or T2D (*p* < 0.05). Pre‐surgery FGF21 concentrations were negatively correlated with the percentage of excess weight loss and the percentage of fat loss.

**Conclusions:**

GDF15 and FGF21 exhibit a different behaviour after Roux‐en‐Y‐gastric bypass surgery, with FGF21 being more closely associated with glycemic status and weight loss. Elevated pre‐surgery FGF21 concentrations could predict a higher difficulty in losing the excess weight after surgery.

## Graphical abstract

Our research shows different post‐surgical patters in GDF15 and FGF21 concentrations following Roux‐en‐Y gastric bypass. GDF15 levels increase significantly in patients with obesity and normoglycemia and in those with obesity and impaired glucose tolerance or type 2 diabetes, while FGF21 concentrations significantly decrease in patients with obesity and impaired glucose tolerance or type 2 diabetes. Moreover, elevated pre‐surgery FGF21 levels may serve as a prognostic biomarker for predicting greater difficulty in losing excess weight after bariatric surgery.

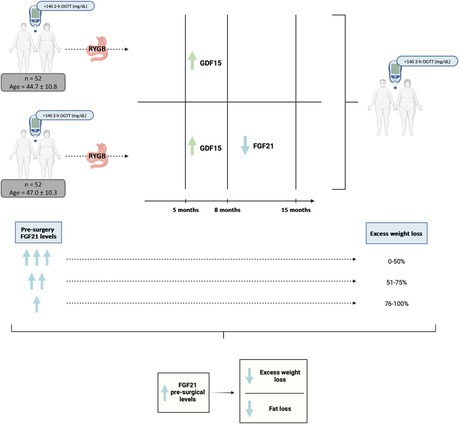

## 1 INTRODUCTION

The alarming increase in obesity worldwide during the last decades has driven a rapid increase of type 2 diabetes (T2D), one of its most related comorbidities.^1^, ^2^ In this sense, it has been demonstrated that achieving at least a 5%–10% weight loss leads to an improvement in obesity‐associated comorbidities and consequently, to a better quality of life.^3^, ^4^, ^5^ Although lifestyle modification is usually the first choice for obesity management, the most recommended approach is a multimodal treatment strategy which may include, in addition to the improvement of eating habits and physical activity, pharmacological therapy.^6^ Usually, when these methods fail, bariatric surgery can be considered.^6^, ^7^ Not only for its ability to induce substantial weight loss, but also for its potential to improve metabolic and cardiovascular outcomes. For instance, bariatric surgery has been shown to be related to alterations in adipokines and to influence endocannabinoid levels, highlighting that bariatric procedures can modulate multiple biological systems that contribute to improved metabolic health.^8^, ^9^ However, although treating obesity has many benefits, predicting the response of a patient to an intervention is difficult, since many clinical factors, including age, gender, body mass index (BMI), ethnicity or the type of bariatric surgery used, influence the outcome.^10^ Hence, it is necessary to identify new prognostic biomarkers that allow predicting the evolution of each patient after a specific intervention, as well as to elucidate the mechanistic explanation for the fat loss and metabolic improvement after the intervention. This will favour the selection of the most appropriate management option in order to personalize the treatment for each patient.

In this sense, the important relationship of growth differentiation factor 15 (GDF15) and fibroblast growth factor 21 (FGF21) with metabolic regulation has exponentially increased the study of both proteins in relation to obesity and its comorbidities.^11^, ^12^, ^13^ GDF15 and FGF21 are stress‐responsive cytokines whose levels are increased in several diseases, including obesity and T2D, being most of them age‐related disorders.^13^, ^14^, ^15^ GDF15 is a transforming growth factor β superfamily member which mediates its effects by interacting with the glial cell‐derived neurotrophic factor family receptor α‐like, mainly located in the hindbrain.^16^, ^17^, ^18^, ^19^ GDF15 regulates energy balance by suppressing food intake^20^, ^21^ or by enhancing energy expenditure in muscle during caloric restriction.^22^ However, there are few and inconsistent data about changes in GDF15 levels after bariatric surgery. While an increase in GDF15 concentrations one year following Roux‐en‐Y gastric bypass (RYGB)^23^, ^24^ and sleeve gastrectomy (SG)^25^ has been found, other authors have recently observed a decrease in GDF15 levels one year after RYGB.^26^

FGF21 is a fibroblast growth factor (FGF) family member, which binds to the FGFR receptor and its β‐klotho co‐receptor to activate the FGFR signalling activity.^27^ FGF21 has been mainly associated with the regulation of energy balance by enhancing energy expenditure.^13^ Moreover, there exists also controversial information about its changes after bariatric surgery. Some data show that FGF21 levels increase after bariatric surgery,^28^, ^29^, ^30^ but other data have not observed significant differences.^15^, ^31^ Furthermore, there are differences in FGF21 levels after bariatric surgery depending on the type of intervention^15^, ^32^ and the follow‐up period.^33^ In line with the increase observed in FGF21 levels after bariatric surgery in some studies, short‐term plasma FGF21 response after surgery could be an important factor to predict an early weight loss.^30^

Therefore, following the hypothesis that GDF15 and FGF21 levels could be related to the improvements of obesity‐related conditions and the resolution of T2D after bariatric surgery, this study aimed to assess the impact of RYGB on their circulating levels as well as their possible relation to weight and fat loss.

## 2 MATERIALS AND METHODS

### 2.1 Study population and experimental design

A total of 104 White patients [52 with obesity and normoglycemia (OB‐NG), 39 with obesity and impaired glucose tolerance (OB‐IGT) and 13 with obesity and type 2 diabetes (OB‐T2D) aged 46 ± 11 y (in the range between 21–70 y), with 69% females] attending the Obesity Unit at the Clínica Universidad de Navarra were selected to analyse the possible changes in GDF15 and FGF21 levels after weight loss induced by RYGB. FGF21 analysis was not possible in all patient samples due to an insufficient volume of plasma (*n* = 37 for the NG group, *n* = 20 for the IGT&T2D group). Patients received oral supplements of vitamins and micronutrients to address potential deficiencies in their post‐bariatric intake and absorption. For the preoperative analysis, samples were collected either on the day of surgery or during the preceding days. Follow‐up revisions were conducted between 5 and 15 months post‐surgery. This follow‐up period was selected to capture the phase with the most pronounced metabolic and physiological changes following bariatric surgery but avoiding the immediate post‐surgical period where changes may be more influenced by acute adaptations. Additionally, this range minimises the variability introduced by long‐term lifestyle changes that may occur later.^34^ Given the reduced number of patients with T2D, those with IGT and T2D were merged into one group, following the approach used in previous studies.^35^ Patients were newly diagnosed with altered glucose homeostasis and, therefore, did not receive anti‐diabetic treatments either before or after bariatric surgery. Glycemic status and remission of T2D were defined according to the American Diabetes Association Criteria.^36^

The inclusion criteria consisted of males and females aged 18–70 y, with a BMI ≥30.0 kg/m^2^ and the absence of psychiatric disorders. The exclusion criteria included severe systemic disease unrelated to obesity, infectious diseases, advanced nephropathy, pregnancy or lactation, eating disorders, cancer, and individuals whose freedom is restricted by legal or administrative requirements. The experimental design was approved by the Universidad de Navarra's Ethical Committee (2021.123) and the study was carried out in accordance with the Declaration of Helsinki. All the patients signed a written informed consent.

### 2.2 Anthropometric and body composition measurements

Body weight was assessed using a digital scale, accurate to 0.1 kg, while the height was determined using a Holtain stadiometer (Holtain Ltd., Crymych, UK), accurate to 0.1 cm. BMI was determined using the formula: BMI = weight (kg)/height (m^2^). Body fat was measured by air‐displacement plethysmography (Bod‐Pod®, COSMED, Rome, Italy) and body fat percentage (BF%) was calculated as previously described.^37^ Waist circumference was measured with a non‐elastic tape positioned between the iliac crest and the rib cage. Blood pressure was measured at the right upper arm using a sphygmomanometer after a 5‐min rest period, with the patient in a semi‐sitting position. The physical activity levels (PAL), defined as the ratio of energy expenditure to basal metabolic rate, were measured following the two‐question questionnaire protocol proposed by Johansson and Westerterp.^38^ The percentage of excess weight loss (%EWL) was calculated based on the ideal body weight (IBW) according to the formula: %EWL = (weight loss*100)/(weight‐IBW). The IBW was calculated using the formula: IBW = 22*(height).^2^, ^39^ The percentage of fat loss (%FATLOSS) was calculated according to the formula: %FATLOSS = 100‐(Final BF%*100/Initial BF%).

### 2.3 Analytical measurements

Biochemical assays were performed as previously reported.^37^, ^40^, ^41^ An enzyme‐amplified chemiluminescence assay (Immulite®, Diagnostic Products, Los Angeles, CA, USA) was used to measure insulin levels, while glucose concentrations together with alanine aminotransferase (ALT) and aspartate aminotransferase (AST) were assessed by an automated analyser (Hitachi Modular P800, Roche, Basel, Switzerland). Total cholesterol and triglyceride levels were determined by enzymatic spectrophotometric methods (Roche). The homeostatic model assessment (HOMA), the quantitative insulin sensitivity check index (QUICKI), and the triglycerides and glucose index were calculated to estimate insulin resistance or insulin sensitivity. The ALT/AST ratio was used as an indicator of hepatic disorders.^42^

Commercially available ELISA kits were used to measure plasma levels of GDF15 (DGD150, R&D Systems, Minneapolis, MN, USA), FGF21 (RD191108200R, BioVendor, Brno, Czech Republic), leptin (RD191001100, BioVendor) and adiponectin (RD191023100, BioVendor) following the manufacturer's protocols. The intraassay coefficients of variation were 2.3%, 2.0%, 5.9% and 4.9%, respectively, while the interassay coefficients of variation were 5.4%, 3.3%, 5.6% and 6.7%, respectively.

### 2.4 Statistical Analysis

Data are presented as mean ± standard deviation (SD). The normal distribution of the data was assessed using the Kolmogorov–Smirnov test. To assess the differences between groups, two‐tailed unpaired or paired Student's *t* tests were performed. Data analysis was conducted with SPSS version 25 (IBM SPSS Statistics, Chicago, IL, USA) and graphs were created using GraphPad Prism 10.0 software (San Diego, CA, USA). A *p* value of <0.05 was regarded as statistically significant.

## 3 RESULTS

### 3.1 GDF15 levels increase after bariatric surgery, whereas FGF21 levels decrease depending on the glycemic status

**FIGURE 1:**
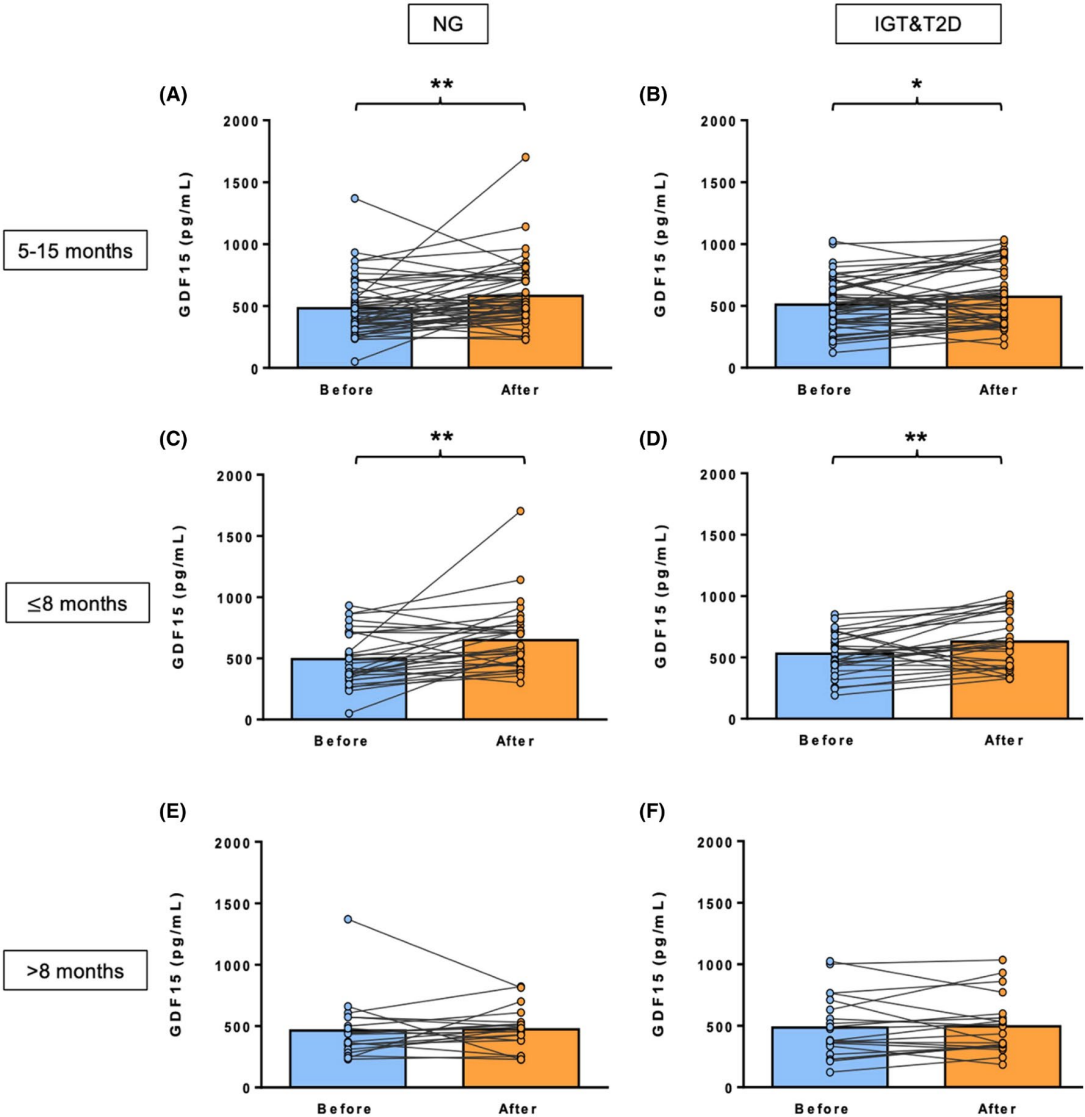
Plasma GDF15 levels before and after bariatric surgery analysed between 5 to 15 months post‐surgery in patients with obesity (A) and NG (*n* = 52) or (B) IGT&T2D (n = 52) and analysed before 8 months post‐surgery in patients with obesity and (C) NG (*n* = 32) or (D) IGT&T2D (*n* = 30) or analysed more than 8 months after surgery in patients with obesity and (E) NG (*n* = 20) or (F) IGT&T2D (*n* = 22). Statistical differences between groups were analysed by two‐tailed paired Student's *t*‐test. *p < 0.05 and **p < 0.01. NG, normoglycemia; IGT, impaired glucose tolerance; T2D, type 2 diabetes.

**FIGURE 2:**
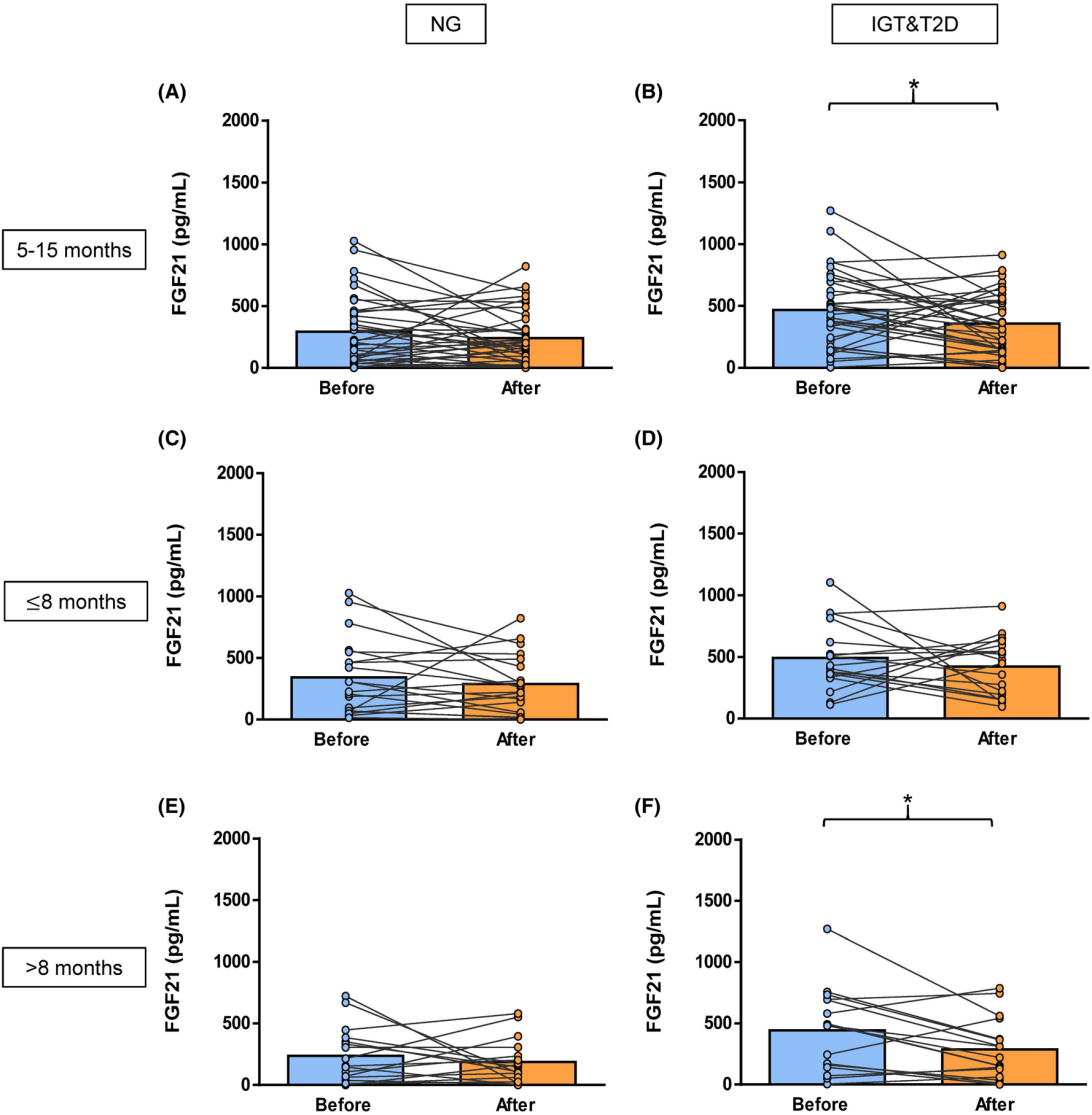
Plasma FGF21 levels before and after bariatric surgery analysed between 5 to 15 months post‐surgery in patients with obesity (A) and NG (*n* = 37) or (B) IGT&T2D (n = 20) and analysed before 8 months post‐surgery in patients with obesity and (C) NG (*n* = 18) or (D) IGT&T2D (*n* = 17) or analysed more than 8 months after surgery in patients with obesity and (E) NG (n = 20) or (F) IGT&T2D (*n* = 21). Statistical differences between groups were analysed by two‐tailed paired Student's *t*‐test. **p < 0.01. NG, normoglycemia; IGT, impaired glucose tolerance; T2D, type 2 diabetes.

Clinical features of people submitted to bariatric surgery are shown in Table 1. Before bariatric surgery, the IGT&T2D group showed similar anthropometric and body composition values compared to the NG group, although a higher waist‐to‐hip ratio in the IGT&T2D group was found (*p* < 0.05). Regarding biochemical variables, the IGT&T2D group exhibited significantly higher glucose (*p* < 0.001), insulin (*p* < 0.01), HOMA (*p* < 0.05) and triglycerides and glucose index levels (*p* < 0.05), while showing a significantly lower QUICKI index (*p* < 0.01), ALT/AST ratio (*p* < 0.05) and adiponectin (*p* < 0.05) concentrations compared to the NG group. Moreover, FGF21 concentrations were significantly higher (*p* < 0.05) in the IGT&T2D group, whereas no significant differences were observed in GDF15 levels between the groups. Between 5 to 15 months after bariatric surgery, significant improvements in several anthropometric measurements such as body weight, BMI, BF%, waist and hip circumferences, systolic and diastolic blood pressure, as well as in some biochemical variables including glucose, insulin, triglycerides, total cholesterol and QUICKI were observed in both groups (all *p* < 0.001) (Table 1). Only the IGT&T2D group showed a significant improvement in HDL‐cholesterol levels after bariatric surgery (*p* < 0.01). In addition, adiponectin concentrations remained significantly higher in the NG group (*p* < 0.05) compared to the IGT&T2D group after surgery, while leptin levels significantly decreased after bariatric surgery in both the NG and the IGT&T2D groups (*p* < 0.001 for both), with no significant difference between groups. This was accompanied by an increase in the PAL value in both groups, with a significantly greater increase in the IGT&T2D group (*p* < 0.001) compared to the NG group (*p* < 0.05). All the patients with T2D achieved diabetes remission. Interestingly, plasma GDF15 levels were significantly increased in both groups after surgery (Table 1; Figure 1A,B), while plasma FGF21 levels decreased slightly in both groups (Table 1; Figure 2A,B), with the decrease being significant only in the IGT&T2D group. A positive correlation was found between pre‐surgical HOMA levels and GDF15 concentrations both before (*p* < 0.01, r = 0.44) and after (*p* < 0.01, r = 0.40) surgery, as well as between post‐bariatric HOMA levels and FGF21 concentrations after surgery (*p* < 0.05, r = 0.46).

**TABLE 1:** Anthropometric and biochemical characteristics of patients submitted to bariatric surgery.

	NG		IGT & T2D		*p*	
Variable	Before RYGB	After RYGB	Before RYGB	After RYGB	Before RYGB	After RYGB
*n*	52	52	52	52	‐	‐
Age (years)	44.7 ± 10.8	45.7 ± 10.8	47.0 ± 10.3	47.8 ± 10.3	‐	‐
Body weight (kg)	119.9 ± 26.6	80.2 ± 16.5***	119.7 ± 22.2	83.2 ± 16.8***	0.967	0.371
Height (m)	1.66 ± 0.09	1.66 ± 0.09	1.66 ± 0.11	1.66 ± 0.11	0.930	0.930
BMI (kg/m^2^)	43.0 ± 6.7	28.9 ± 4.8***	43.3 ± 5.7	30.0 ± 4.8***	0.815	0.214
Body fat (%)	52.7 ± 5.7	36.0 ± 9.3***	51.0 ± 6.7	35.1 ± 9.8***	0.172	0.631
Waist circumference (cm)	124 ± 14	94 ± 14***	125 ± 13	98 ± 12***	0.515	0.213
Hip circumference (cm)	133 ± 15	106 ± 11***	130 ± 11	107 ± 11***	0.200	0.644
WHR	0.93 ± 0.09	0.89 ± 0.09**	0.98 ± 0.09	0.91 ± 0.08***	**<0.05**	0.201
WHtR	0.73 ± 0.13	0.57 ± 0.08***	0.76 ± 0.06	0.58 ± 0.07***	0.203	0.244
SBP (mm Hg)	131 ± 15	113 ± 13***	136 ± 20	119 ± 16***	0.142	0.085
DBP (mm Hg)	82 ± 10	71 ± 10***	85 ± 12	71 ± 15***	0.152	0.947
Glucose (mg/dL)	94 ± 12	85 ± 7***	110 ± 22	87 ± 20***	**<0.001**	0.477
Glucose 2‐h OGTT (mg/dL)	112 ± 18	‐	187 ± 33	‐	**<0.001**	‐
Insulin (μ U/mL)	18.6 ± 13.3	6.5 ± 3.4***	22.4 ± 10.8	7.4 ± 4.1***	0.139	0.300
Insulin 2‐h OGTT (μ U/mL)	97.6 ± 48.1	‐	145.9 ± 73.7	‐	**<0.01**	‐
HOMA	4.43 ± 3.35	1.34 ± 0.72***	6.28 ± 3.76	1.59 ± 0.98***	**<0.05**	0.201
QUICKI	0.32 ± 0.03	0.38 ± 0.39***	0.30 ± 0.02	0.37 ± 0.04***	**<0.01**	0.299
TyG index	8.6 ± 0.5	8.0 ± 0.4***	8.8 ± 0.4	8.1 ± 0.4***	**<0.05**	0.387
Triglycerides (mg/dL)	124.2 ± 67.8	76.7 ± 25.5***	128.5 ± 56.4	74.5 ± 29.4***	0.746	0.712
Cholesterol (mg/dL)	196.4 ± 32.2	161.3 ± 31.7***	198.9 ± 31.8	159.4 ± 42.4***	0.717	0.813
LDL‐cholesterol (mg/dL)	117.2 ± 30.0	90.4 ± 26.6***	121.3 ± 26.9	86.6 ± 28.7***	0.493	0.524
HDL‐cholesterol (mg/mL)	54.1 ± 16.3	54.7 ± 13.5	48.9 ± 11.4	54.3 ± 15.8**	0.081	0.896
Uric acid (mg/dL)	5.67 ± 1.41	4.49 ± 1.31***	5.67 ± 1.40	4.31 ± 1.40***	0.998	0.537
ALT (IU/L)	22.5 ± 14.0	22.2 ± 15.4	27.9 ± 15.8	27.1 ± 19.5	0.090	0.170
AST (IU/L)	17.5 ± 10.6	21.4 ± 16.6	18.9 ± 8.6	21.6 ± 13.0	0.479	0.952
AST/ALT ratio	0.92 ± 0.26	1.02 ± 0.33	0.74 ± 0.21	0.90 ± 0.20**	**<0.05**	0.090
GGT (IU/L)	21.3 ± 14.2	12.8 ± 10.8***	28.0 ± 19.2	26.5 ± 53.9	0.188	0.201
PAL	1.54 ± 0.11	1.60 ± 0.98*	1.51 ± 0.12	1.63 ± 0.12***	0.453	0.689
GDF15 (pg/mL)	482.4 ± 225.9	582.0 ± 253.1**	511.6 ± 205.7	574.6 ± 233.0*	0.495	0.877
FGF21 (pg/mL)	293.9 ± 274.6	241.7 ± 214.8	468.1 ± 298.6	357.2 ± 246.5*	**<0.05**	0.888
Adiponectin (μ g/mL)	11.4 ± 5.6	17.9 ± 7.5***	9.1 ± 4.3	15.0 ± 6.4***	**<0.05**	**<0.05**
Leptin (ng/mL)	79.0 ± 39.4	22.2 ± 23.5***	72.9 ± 42.0	25.8 ± 28.1***	0.447	0.485
Adipoq/Lep ratio	0.18 ± 0.14	1.34 ± 1.24***	0.19 ± 0.18	1.63 ± 2.00***	0.800	0.393

*Note*: Data are mean ± SD. Statistical differences between groups were analysed by two tailed unpaired or paired Student's *t*‐tests to compare differences between groups or before and after surgery, respectively. *p < 0.01, **p < 0.01 and ***p < 0.001 versus before bariatric surgery in each group. Significant differences are highlighted in bold.

Abbreviations: Adipoq, adiponectin; Lep, leptin; ALT, alanine aminotransferase; AST, aspartate aminotransferase; BMI, body mass index; DBP, diastolic blood pressure; GDF‐15, growth differentiation factor 15; GGT, γ‐glutamyl transferase; HOMA, homeostatic model assessment; IGT; impaired glucose tolerance, NG, normoglycemia; PAL, physical activity level; QUICKI, quantitative insulin sensitivity check index; SBP, systolic blood pressure; T2D, type 2 diabetes; TyG index, triglycerides and glucose index; WHR, waist‐to‐hip ratio; WHtR, waist‐to‐height ratio.

Due to the wide range of time between surgery and the analysis of GDF15 and FGF21 concentrations after surgery among patients (5–15 months), the study groups were subclassified according to post‐surgical time, to further explore whether the response in these molecules might be time dependent. The cohort was split into those patients whose plasma samples were analysed between 5 and 8 months after surgery (6.5 months of average for the NG group and 6.2 months for the IGT&T2D group) and those whose plasma samples were analysed between 8 and 15 months after surgery (10.6 months for the NG group and 11.0 months for the IGT&T2D group). In the people analysed up to 8 months post‐bariatric surgery, a significant increase in plasma GDF15 levels in the NG and IGT&T2D groups was found (Figure 1C,D). Nevertheless, in those patients analysed more than 8 months after surgery, no significant changes in plasma GDF15 levels were observed (Figure 1E,F), suggesting that the initial increase in GDF15 levels after surgery returned to pre‐surgical levels over time. In relation to FGF21, the NG subgroups showed a slight non‐significant decrease in their levels (Figure 2C,D). Despite the IGT&T2D subgroups showing the same tendency, the decrease in FGF21 levels was significant in the group analysed 8 months after surgery (*p* < 0.05) (Figure 2E,F), suggesting that FGF21 levels could be involved in the T2D resolution.

### 3.2 Lower FGF21 levels before bariatric surgery are related to a higher excess weight loss

**FIGURE 3:**
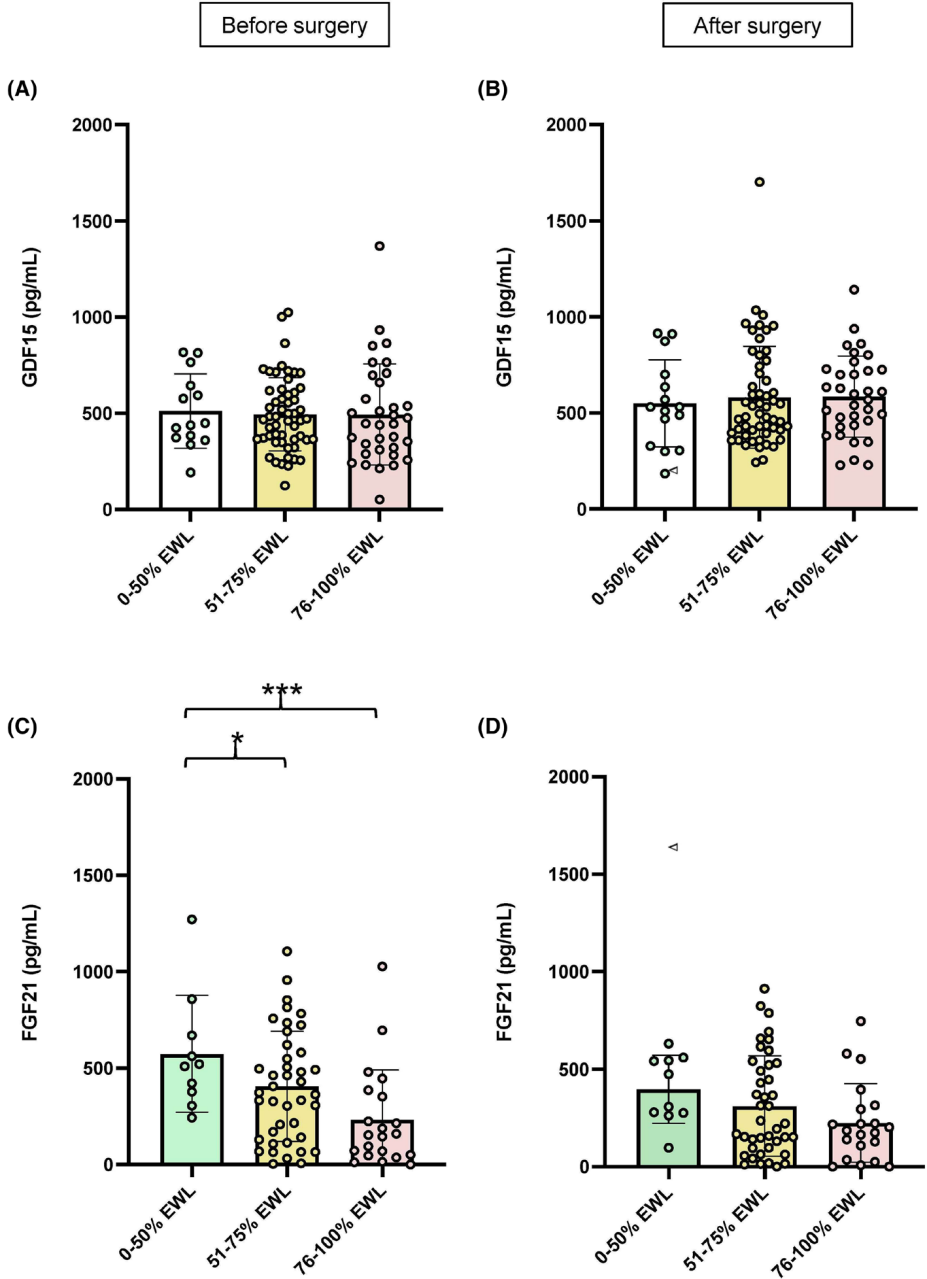
Plasma GDF15 levels in relation to the %EWL before (A) and after (B) bariatric surgery. Plasma FGF21 levels in relation to the %EWL before (C) and after (D) bariatric surgery. *p < 0.05. %EWL, percentage of excess weight loss.

**FIGURE 4:**
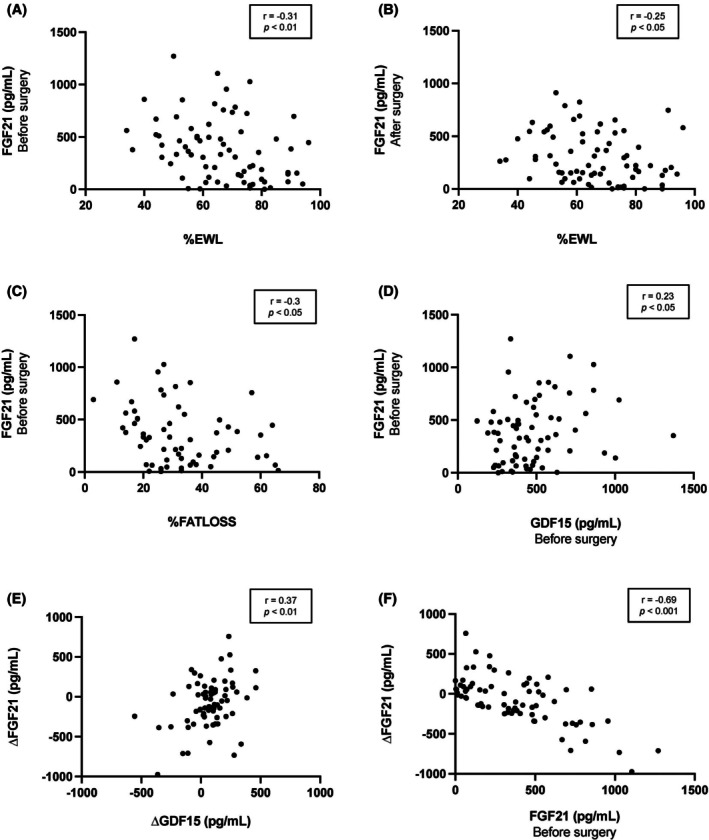
Scatter diagrams showing the correlation between (A) FGF21 levels before surgery and %EWL, (B) FGF21 levels after surgery and %EWL, (C) FGF21 levels before surgery and %FATLOSS, (D) FGF21 and GDF15 levels before surgery, (E) changes in FGF21 and GDF15 levels after surgery and (F) changes in FGF21 levels after surgery and FGF21 levels before surgery. %EWL, percentage of excess weight loss; %FATLOSS, percentage of fat loss.

To further analyse a possible association between changes in GDF15 and FGF21 levels after bariatric surgery and weight loss, the patients were classified into three groups considering the amount of EWL after surgery. No significant differences were found in GDF15 levels before and after bariatric surgery in relation to %EWL (Figure 3A,B). By contrast, patients with higher %EWL exhibited significantly lower levels of FGF21 before RYGB (Figure 3C). This significant difference disappeared after surgery (Figure 3D). In this regard, a negative correlation between FGF21 levels and %EWL before (Figure 4A) and after (Figure 4B) bariatric surgery was found, and plasma FGF21 concentrations before surgery also showed a negative correlation with the percentage of fat loss (%FATLOSS) (Figure 4C). On the opposite, pre‐surgical and post‐surgical GDF15 levels did not show any correlation with the %EWL (r = −0.05, *p* = 0.620; r = −0.01, *p* = 0.976; respectively) or the %FATLOSS (r = 0.08, *p* = 0.464; r = −0.01, *p* = 0.976 respectively). Moreover, a positive correlation between GDF15 and FG2F1 levels before surgery was observed (Figure 4D), although this correlation disappeared after surgery (r = 0.02, *p* = 0.863). In addition, a positive association between changes in GDF15 and changes in FGF21 was found (Figure 4E), together with a negative correlation between changes in FGF21 concentrations and FGF21 levels before surgery (Figure 4F).

## 4 DISCUSSION

The obesity pandemic has increased the emergency to find effective anti‐obesity therapies along with better biomarkers for diagnosis and prognosis of obesity and its associated comorbidities.^6^, ^43^ In this context, our findings show a different behaviour of GDF15 and FGF21 concentrations after bariatric surgery, with FGF21 levels being more related to weight loss after surgery. These results suggest that FGF21 concentrations could be a potential biomarker to predict weight loss after bariatric surgery.

Regarding GDF15, a rise in its plasma levels after RYGB was observed, which is consistent with previous studies.^23^, ^24^, ^25^, ^26^ Nevertheless, the increase was not maintained over time, being only significant in the samples analysed between 5 to 8 months after bariatric surgery. This suggests that the initial increase in GDF15 levels would be attenuated over time. These results are consistent with those reported by Schmid et al.^44^ who found an increase in GDF15 levels 3 months after RYGB surgery, followed by a decrease in GDF15 concentrations below baseline levels 6 months post‐surgery. To explain the changes observed in GDF15 levels, three possibilities were considered. Firstly, the stress caused by RYGB surgery may lead to an increase in GDF15 levels, which could facilitate the early resolution of T2D and the improvement of obesity alterations following bariatric surgery, then returning to presurgical levels. Secondly, the increase in GDF15 levels caused by the stress of RYGB surgery could be maintained over time due to a state of GDF15 resistance or reduced sensitivity in people living with obesity. After the improvement in the anthropometric variables and body composition achieved by bariatric surgery, this resistance could be reduced, leading to a decrease in GDF15 levels. At this point, GDF15 could help to improve anthropometric and body composition measures. Thirdly, another potential factor contributing to the sustained elevation of GDF15 levels could be the energy imbalance resulting from the reduced caloric intake induced by bariatric surgery. Through unknown mechanisms, GDF15 levels remain elevated to promote weight loss, helping to maintain a negative energy balance, subsequently returning to baseline levels once the metabolic improvement has been achieved. Although further studies are necessary to discern between these possibilities, this potential association of GDF15 with the improvement of obesity and T2D might be related to its role in metabolic homeostasis by suppressing food intake, favouring weight loss.^12^ Regarding FGF21, the IGT&T2D group showed significantly higher FGF21 concentrations compared to the NG group before bariatric surgery, in agreement with previous results from our group.^15^ The IGT&T2D group also exhibited significantly higher levels of variables such as fasting glucose, 2‐h OGTT glucose, 2‐h OGTT insulin and HOMA, along with lower levels of QUICKI, all of which are related to insulin resistance, compared to the NG group. This is consistent with the strong relationship between FGF21 and glycemic status.^13^ Therefore, the elevated FGF21 concentrations observed in the IGT&T2D group may reflect a compensatory mechanism to improve insulin resistance by stimulating glucose uptake. This idea is supported by the significant decrease in FGF21 levels after surgery in the IGT&T2D group, along with the decrease in fasting glucose and HOMA levels, as well as with the increase in QUICKI levels. Consequently, the absence of differences in FGF21, fasting glucose, HOMA and QUICKI levels between the NG and IGT&T2D groups post‐surgery could be mediated by the glycemic status improvement in the IGT&T2D group. The slight decrease observed in FGF21 levels in the IGT&T2D group was opposed to what was observed in previous studies.^28^, ^29^ Differences in the follow‐up period could explain the diverse findings compared to other studies. Other studies have shown an increase in FGF21 levels within 3 months after bariatric surgery, which then returned to baseline levels or even under baseline levels.^30^, ^31^ Since our data cover the period from 5 to 15 months after bariatric surgery, we were unable to observe the possible early increase in FGF21 levels that might take place during the previous months. This information, along with our findings, indicates that the FGF21‐associated response could be faster than the GDF15 response, whose levels remain elevated for a longer period. This might explain why we did not observe an increase in FGF21 levels after surgery. Furthermore, the decrease found in FGF21 levels was not significant in the NG group, while in the IGT&T2D group, FGF21 levels significantly decreased from 8 to 15 months after bariatric surgery. This suggests that the resolution of T2D leads to a significant decrease in FGF21 levels, unlike in the NG group, highlighting the strong relationship between FGF21 and the glycemic status due to the role of FGF21 in improving insulin sensitivity.^13^ This might shed light on a previous study from our group^15^ where we did not find significant differences in FGF21 levels after RYGB surgery since the groups were not split based on their glycemic status. However, consistent with the difference between the NG and the IGT&T2D groups, that study found a positive correlation between changes in FGF21 levels and changes in HOMA. Moreover, we found a positive correlation between FGF21 and HOMA levels after surgery, which emphasizes the relation between FGF21 and the glycemic status.

Unlike GDF15, FGF21 levels showed a significant negative correlation with %EWL and %FATLOSS, suggesting that high levels of FGF21 before RYGB could be indicative of a worse response to the surgery. In this context, a recent article of De Luca et al.^30^ demonstrated that the magnitude of the increase in FGF21 levels 3 months after bariatric surgery appears to be linked to a greater weight loss during the first year following the surgery. Likewise, a negative correlation between pre‐surgical FGF21 levels and the change of FGF21 concentrations after surgery was found. Therefore, in accordance with their results, we observed that patients with higher initial levels of FGF21 were those who had a smaller post‐surgical increase in FGF21 levels and a smaller weight loss.

Moreover, a positive association between the changes in FGF21 and GDF15 levels after bariatric surgery was found, suggesting a similar regulatory mechanism for both cytokines following the surgery, with some time differences that need further investigation. More studies are required to understand whether these changes contribute to the improvement of metabolic alterations after bariatric surgery or if they are not essential for the benefits observed, as it was previously shown in mice,^45^, ^46^ and to analyse whether a possible feedback loop between GDF15 and FGF21 exists. Additionally, it would be of interest to explore in future studies variations in the levels of FGF21 and GDF15, considering obesity and severe obesity as separate disease entities, as it has been shown that their pathophysiology can differ significantly.^47^, ^48^ Therefore, variations in the levels of GDF15 and FGF21 after bariatric surgery could also be observed between these groups.

The study has some limitations. Firstly, all participants were White people, and it would be necessary to study whether our conclusions could be extended to other populations. Secondly, further studies in larger cohorts and with longer follow‐up periods would be necessary. However, the appropriate characterization and homogeneity of the population, including only patients who underwent RYGB, reduce variability associated with different bariatric procedures and enable robust conclusions to be obtained in our study.

## 5 CONCLUSION

Taken together, FGF21 levels decrease earlier than GDF15 following bariatric surgery, and FGF21 is more associated with the glycemic status and the fat loss than GDF15. Moreover, to our knowledge, this is the first study demonstrating that elevated levels of FGF21 before bariatric surgery could predict a higher difficulty in losing excess weight. Further studies would be necessary to understand the implications of the changes in FGF21 and GDF15 after bariatric surgery, which follow a different pattern, in relation to the improvement of obesity and the resolution of T2D.

In summary, the identification of FGF21 as a potential preoperative biomarker for weight loss offers a promising approach for optimizing treatment strategies in patients with obesity. This could lead to more individualized treatment plans for obesity management. Patients with higher FGF21 levels could be considered for other nutritional, behaviouralbehavioral or pharmacological interventions, either before or after surgery, to enhance the weight loss achievement.

## AUTHOR CONTRIBUTIONS

L.S.‐G., G.F. and J.G.‐A. designed the study and obtained the funds. L.S.‐G., G.F. and J.G.‐A. analysed and interpreted the data. L.S.‐G. and J.G.‐A. drafted the manuscript. L.S.‐G., V.C., B.R., M.A.‐A, A.R., S.B., V.V., R.M., C.M.P., C.S., J.E., G.F., and J.G.‐A. provided study materials or performed experiments. L.S.‐G., V.C., B.R., A.R., S.B., V.V., R.M., C.S., J.E., G.F., and J.G.‐A. critically revised the article for important intellectual content. All authors read and approved the final version of the article. L.S.‐G., G.F. and J.G.‐A. are the guarantors of this work and, as such, had full access to all the data in the study and take responsibility for the integrity of the data and the accuracy of the data analysis.

## CONFLICT OF INTEREST STATEMENT

GF declares payment of honoraria for lectures from Novo Nordisk as a member of the OPEN Spain Initiative as well as payment of honoraria for attendance at advisory boards from Lilly, Novo Nordisk, Regeneron, and AstraZeneca and declares no competing interest, financial or nonfinancial, related to this work. The other authors declared no conflict of interest.

## Data Availability

The datasets used and analysed during the current study are available from the corresponding author on reasonable request.
